# Mucormycosis (black fungus) infection and misuse of corticosteroids in COVID 19 patients in Syria

**DOI:** 10.1016/j.amsu.2022.103463

**Published:** 2022-03-09

**Authors:** Aya Yaser Mohsen, Jollanar Ghanem Mohammad, Moatasem Hussein Al-janabi

**Affiliations:** Tishreen University Faculty of Medicine, Lattakia, Syria; Tishreen University Faculty of Medicine, Lattakia, Syria; Department of Pathology, Cancer Research Center, Tishreen University Hospital, Lattakia, Syria

**Keywords:** Mucormycosis, Black fungus, COVID-19, Corticosteroids

Dear Editor,

Mucormycosis (also known as zygomycosis) is a serious and rare fungal infection, which observed in a number of COVID-19 cases recently. Mucormycosis is found throughout the environment, mostly in the soil [1,2]. Mucormycosis is an aggressive infection that usually affects immunocompromised conditions, poorly managed diabetics, and COVID-19 patients who are on steroid therapy on a long-term basis such as dexamethasone [3,4]. Generally, Mucormycosis spores are transmitted in several ways, especially by inhaling from the air to our sinuses, causing many symptoms like fever, headache, nasal congestion, and the most important sign is “black lesions” that quickly become more severe. Also, it may damage the lung, skin, digestive system, brain, and up to death [1,2]. For the serious complications of mucormycosis infection, we would highlight in this paper, the relationship between this infection and the misuse of steroids in our country. Syria is suffering from the war since 2011, and like any cruel war, all the sectors were damaged. In addition, the lack of health education makes people vulnerable to rumors and misconceptions about the medications and how it works. High-dose corticosteroids are used by COVID-19 patients because they thought it would prevent them from the complications of virus corona and keep them safe. Long-term or high doses of systemic corticosteroids especially in COVID-19 patients lead to a wide range of bacterial and fungal infections such as mucormycosis which is associated with a high mortality rate [2,3]. Recently, a lot of cases of mucormycosis are presented to our hospital every day due to overuse or misuse of corticosteroids. Mucormycosis is mainly diagnosed by a sample of fluid from a nose or tissue biopsy and sent to a laboratory [4]. Management principles include avoiding predisposing factors, antifungal medication, and surgery in the advanced stage, which can lead to loss of the jawbone or even the eye [2,5]. Unfortunately, that was mostly happening to Syrian COVID -19 patients because of the case of poverty, destitution, and constant fear of death which make them didn't go to the clinical or hospital until the condition was worsened. In conclusion, ignorance, lack of health awareness regarding the coronavirus and its severity, and uncertainty toward vaccine lead patients to self-medication and abuse of systemic corticosteroids. We recommend establishing awareness campaigns, in which the United Nations Population Fund participated, aimed at introducing the information on the seriousness of coronavirus infection, COVID-19 vaccines including the effectiveness and safety, and the necessary health guidelines for preventing the rampant usage of steroids in patients with COVID-19.

## Ethical approval

No ethical approval was needed.

## Sources of funding

None declared.

## Author contribution

Aya Yaser Mohsen: Participated in writing the letter. Jollanar Ghanem Mohammad: Participated in writing the letter. Moatasem Hussein Al-janabi: Reviewing the letter.

## Conflicts of interest

The authors have no conflicts of interest to declare.

## Registration of research studies

Not applicable.

## Guarantor

Moatasem Hussein Al-janabi.

## Consent

N/A.

## Provenance and peer review

Not commissioned, externally peer reviewed.
